# Dysphagia and its effects on swallowing sounds and vibrations in adults

**DOI:** 10.1186/s12938-018-0501-9

**Published:** 2018-05-31

**Authors:** Joshua M. Dudik, Atsuko Kurosu, James L. Coyle, Ervin Sejdić

**Affiliations:** 1 Department of Electrical and Computer Engineering, University of Pittsburgh, Pittsburgh, PA USA; 2 Department of Communication Sciences and Disorders, University of Pittsburgh, Pittsburgh, PA USA

**Keywords:** Dysphagia, Cervical auscultation, Signal characteristics, Pathology, Swallowing vibrations, Swallowing sounds

## Abstract

**Background:**

To utilize cervical auscultation as a means of screening for risk of dysphagia, we must first determine how the signal differs between healthy subjects and subjects with swallowing disorders.

**Methods:**

In this experiment we gathered swallowing sound and vibration data from 53 (13 with stroke, 40 without) patients referred for imaging evaluation of swallowing function with videofluoroscopy. The analysis was limited to non-aspirating swallows of liquid with either thin (< 5 cps) or viscous ($\approx 300\,{\text{cps}}$) consistency. After calculating a selection of generalized time, frequency, and time frequency features for each swallow, we compared our data against our findings in a previous experiment that investigated identical features for a different group of 56 healthy subjects.

**Results:**

We found that nearly all of our chosen features for both vibrations and sounds showed significant differences between the healthy and disordered swallows despite the absence of aspiration. We also found only negligible differences between dysphagia as a symptom of stroke and dysphagia as a symptom of another condition.

**Conclusion:**

Non-aspirating swallows from healthy controls and patients with dysphagia have distinct feature patterns. These findings should greatly help the development of the cervical auscultation field and serve as a reference for future investigations into more specialized characterization methods.

## Background

Abnormal swallowing, referred to as dysphagia, can present multiple different signs and symptoms such as the feeling of food being stuck in the throat, difficulty in placing and controlling food in the mouth, and coughing after swallowing [1]. These swallowing difficulties can result from a multitude of different medical conditions, particularly neurological conditions, but physical trauma and stroke are among the most prevalent individual causes [2]. Though not immediately life-threatening in all but the most extreme cases, dysphagia that is not treated in a timely manner can lead to serious medical issues such as malnutrition, dehydration, or pneumonia, and so early detection of dysphagia risk is a high priority [2, 3].

The current accepted diagnostic gold-standard methods for evaluating swallowing function include nasopharyngeal endoscopy and videofluoroscopy [4, 5]. However these specialized procedures are not available in all situations, and so attempts have been made to improve the accuracy of simpler and more mobile swallowing screening techniques. Several such methods have been investigated over the years, most notably including the widely adopted 3 ounce water swallow challenge which achieves a very high sensitivity in predicting aspiration [6, 7]. Unfortunately it seriously overestimates the likelihood of aspiration, thereby forcing potentially unpleasant or harmful interventions upon patients waiting for a full diagnostic exam [6, 7]. Screening methods that use more portable instrumentation, such as pulse-oximetry and surface electromyography, have also demonstrated limited sensitivity and specificity [8, 9]. Cervical auscultation on the other hand, which is also an imprecise screening technique when deployed in a traditional manner, has shown some promise attributable to recent technological advances and has been studied in much greater detail [10].

Cervical auscultation, a method by which a clinician uses a stethoscope to listen to the throat while a patient swallows boluses of food and liquid, has not yet demonstrated adequate predictive value for swallowing disorders despite claims to the contrary by numerous methodologically flawed studies [3, 10, 11]. Recently, however, researchers have begun using microphones and accelerometers to obtain data [12–14]. As digital devices, these transducers do not have the same physical or interpretive biases as humans, making it much easier to apply desired signal processing and analysis techniques to the data. Multiple noise filtering as well as feature extraction and classification techniques have been applied in previous studies to improve the signal quality and objectively assess the information obtained [12].

If an improved, non-invasive method of investigating swallowing difficulties is to be developed, it is imperative to characterize and compare swallows from both healthy and non-healthy patients. From there, it could then be possible to determine how these two sets of data differ and subsequently determine how to automatically differentiate the two classes. Accomplishing this normal-abnormal differentiation with greater precision than current methods would be invaluable with regards to the early detection of dysphagia and prevention of subsequent adverse events. Our paper offers two notable contributions. First, mirroring a previous study that investigated only healthy subjects [15], we investigate mathematical features from time, frequency, and time–frequency domains of swallowing vibrations and sounds simultaneously recorded from patients with swallowing disorders. Second, we then compare the values of these features with the swallowing signal features obtained from healthy participants. We hypothesized that, since swallowing performance is known to differ between these two populations, our features would show statistical differences in the resulting vibrations and sounds between our test groups. We analyzed only those swallows that did not result in aspiration because swallows in which the person aspirates rarely occur in healthy people [16, 17] and we sought to determine whether our method of cervical auscultation could differentiate between healthy and disordered swallows.

## Methods

### Equipment

**Fig. 1:**
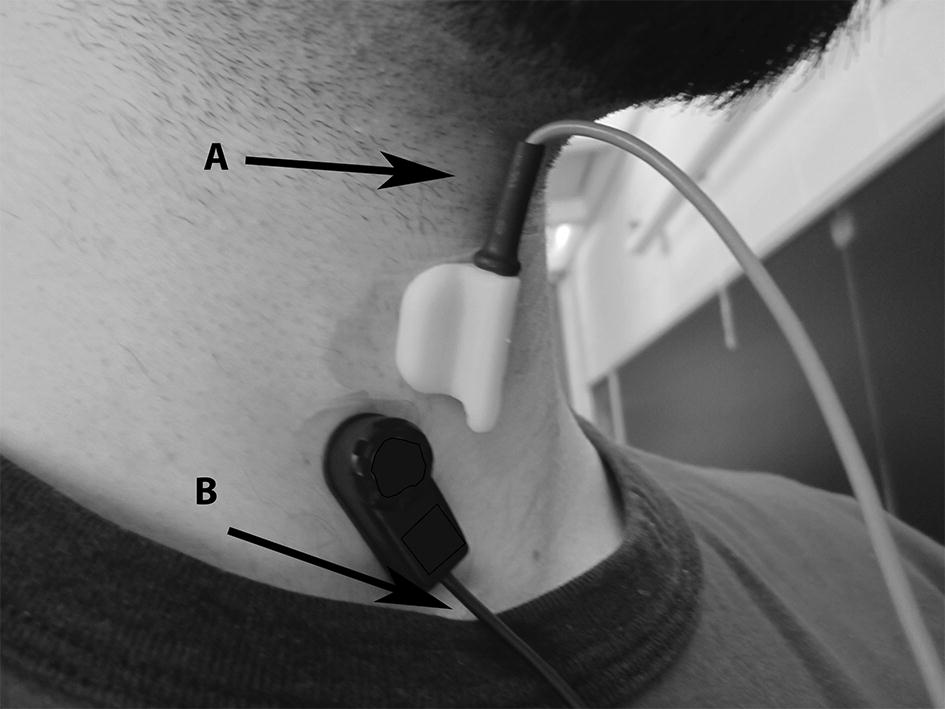
Transducer mounting locations. Location of recording devices during data collection. A: Thyroid cartilage B: top of the suprasternal notch For reference, the microphone (lower device) is approximately 10 × 30 mm and the accelerometer (upper device) is aligned with the centre axis of the neck. This figure has been previously published by BioMed Central in [41]

Our recording equipment consisted of a tri-axial accelerometer and a contact microphone attached to the participant’s neck with double-sided tape. The accelerometer (ADXL 327, Analog Devices, Norwood, Massachusetts) was mounted in a custom plastic case, and affixed over the cricoid cartilage (as seen in Fig. 1) in order to provide the highest signal quality [18]. The two main accelerometer axes were aligned parallel to the front of the neck (approximately parallel to the cervical spine) and perpendicular to the same surface (approximately perpendicular to the coronal plane). These directions will be referred to as superior–inferior (S–I) and anterior–posterior (A–P) axes, respectively. Data from the third axis of the accelerometer was not used in this study. The sensor was powered by a power supply (model 1504, BK Precision, Yorba Linda, California) with a 3V output, and the resulting signals were bandpass filtered from 0.1 to 3000 Hz with ten times amplification (model P55, Grass Technologies, Warwick, Rhode Island) as swallowing vibrations have been shown to be band-limited to approximately this range [19]. The voltage signals for each axis of the accelerometer were fed into a National Instruments 6210 DAQ and recorded at 20 kHz by the LabView program Signal Express (National Instruments, Austin, Texas). This set-up has been proven to be effective at detecting swallowing activity in previous studies by maximizing the signal-to-background-noise ratio [19, 20]. The microphone (model C 411L, AKG, Vienna, Austria) was placed below the accelerometer and slightly towards the right lateral side of the trachea so as to avoid contact between the two sensors but record events from approximately the same location (see Fig. 1). This location has previously been described to be appropriate for collecting swallowing sound signals by maximizing the signal-to-background-noise ratio [21, 22]. The microphone was powered by a power supply (model B29L, AKG, Vienna, Austria) set to ‘line’ impedance with a volume of ‘9’ and the resulting voltage signal was sent to the previously mentioned DAQ. Unlike the swallowing vibrations, this signal was left unfiltered as an upper limit to the bandwidth of swallowing sounds has not yet been found. Instead we recorded the entire dynamic range of our microphone signal (10 Hz–20 kHz) to ensure that we did not lose any important components of our signal. Again, the signal was sampled by Signal Express at 20 kHz. This data recording setup is virtually identical to that found in our previous work [15] so that the data sets could be compared accurately.

For the non-healthy patients only, concurrent videofluoroscopy images were also obtained during their examinations as described in a later section. For these participants, the images output by the x-ray videofluoroscopy machine (Ultimax system, Toshiba, Tustin, CA) were input to a video capture card (AccuStream Express HD, Foresight Imaging, Chelmsford, MA) and recorded with the previously mentioned LabView program.

### Participants and data collection

Data from our control group, healthy subjects without swallowing disorders, was gathered and reported on in a previous study [15]. In that study, a total of 55 healthy participants (28 males, 27 females, mean age 39) were recruited from the neighborhoods surrounding the University of Pittsburgh campus. All healthy participants confirmed that they had no history of swallowing disorders, head or neck trauma or major surgery, chronic smoking, or other conditions which may affect swallowing performance. All testing was performed in the iMED laboratory facilities at the University of Pittsburgh. The healthy participants were presented with chilled (5°) water in five separate 8 mL cups. With their head in a neutral position, they were asked to make five swallows with a few seconds of rest between each swallow while taking no more than a single bolus from each cup. This process was repeated while using Resource Thickened Apple Juice—Moderately Thick 400 (Nestlé Health Care, Inc. Florham Park, NJ) to demonstrate swallowing higher viscosity fluids. These two categories (listed a ‘water swallows’ and ‘honey-thick swallows’ in our previous work [15]) will be referred to as healthy thin swallows and healthy viscous swallows, respectively, in the remainder of this manuscript. A total of 550 unique data points were recorded from healthy subjects where 225 swallows were recorded with each bolus type.

**Fig. 2:**
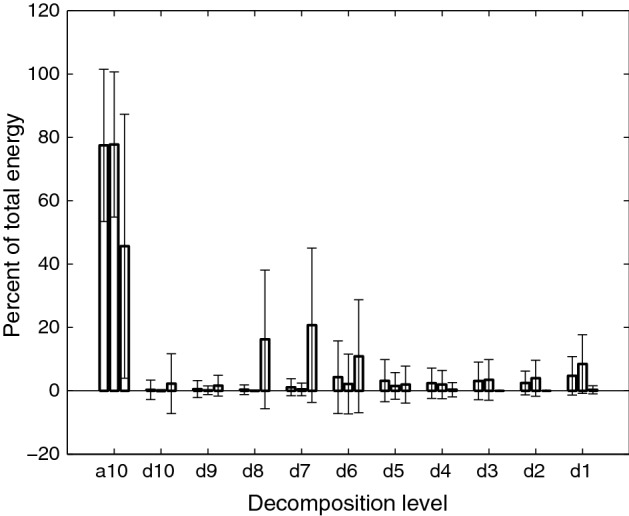
Wavelet energy composition of swallowing vibrations and sounds during thin swallows. From left to right, the bars for each decomposition level correspond to the signals recorded from the anterior–posterior accelerometer, the superior–inferior accelerometer, and the microphone

The non-healthy participants consisted of a separate group of 53 patients with suspected dysphagia that were scheduled to undergo a videofluoroscopic swallowing evaluation at the University of Pittsburgh Medical Center (Pittsburgh, Pennsylvania). Thirteen of these non-healthy patients (10 men, 3 women, mean age 66) had a current diagnosis of stroke while the remaining 40 (24 men, 16 women, mean age 62) had medical conditions unrelated to stroke. Routine, standard institutional screening and clinical assessment procedures were used to identify all patients who were in need of an instrumental examination to evaluate and manage their swallowing disorder. Those patients that had a history of major head or neck surgery, were equipped with assistive devices that obstructed the anterior neck such as a tracheostomy tube, or were not sufficiently competent to give informed consent were not included in the study, but no other conditions were excluded.

Patients with dysphagia did not undergo a standardized data collection procedure, as the videofluoroscopy examination is routinely modified by the examiner to suit the individual patient, but analyzed swallows were limited to those made while in a neutral head position. The liquids swallowed during the examination included chilled (5 °C) Varibar Thin Liquid, with $<5\,{\text{cps}}$ consistency, and Varibar Nectar, with $\approx 300\,{\text{cps}}$ consistency, (Bracco, Milan, ITA) presented as either self-administered from a cup or administered by the examiner from a 5 mL spoon. The consistencies of these two liquids were determined to be sufficiently similar to the liquids presented to healthy participants based on available product information and qualitative guidelines. On average, each patient contributed four swallows that were usable in our analysis, but the exact number varied between one and ten due to the personalized nature of the medical exam. A total of 64 swallows were recorded from non-healthy patients with stroke while 158 were recorded from non-healthy patients without a history of stroke.

### Digital signal processing

The signals recorded with the microphone and accelerometer underwent the same digital processing steps applied in our previous work [15], which we reproduce here for the convenience of the reader. The techniques cited have been developed and optimized for cervical auscultation signals in previous studies.

At an earlier date, the accelerometer’s baseline output was recorded and modified covariance auto-regressive modeling was used to characterize the device noise [23, 24]. The order of the model was determined by minimizing the Bayesian information criterion [23]. These autoregressive coefficients were then used to create a finite impulse response filter and whiten the recording device noise in our signal [23]. Afterwards, motion artifacts and other low frequency noise were removed from the signal through the use of least-square splines. Specifically, we used fourth-order splines with a number of knots equal to $\dfrac{Nf_{l}}{f_{s}}$, where *N* is the number of data points in the sample, *fs* is the original 20 kHz sampling frequency of our data, and $f_{l}$ is equal to either 3.77 or 1.67 Hz for the superior–inferior or anterior–posterior direction, respectively. The values for $f_{l}$ were calculated and optimized in previous studies [25]. After subtracting this low frequency motion from the signal we removed white noise from our data by using tenth-order Meyer wavelets with soft thresholding [26]. The optimal value of the threshold was determined through previous research to be $\sigma \sqrt{2\log N}$, where *N* is the number of samples in the data set and $\sigma$, the estimated standard deviation of the noise, is defined as the median of the down-sampled wavelet coefficients divided by 0.6745 [26].

**Fig. 3:**
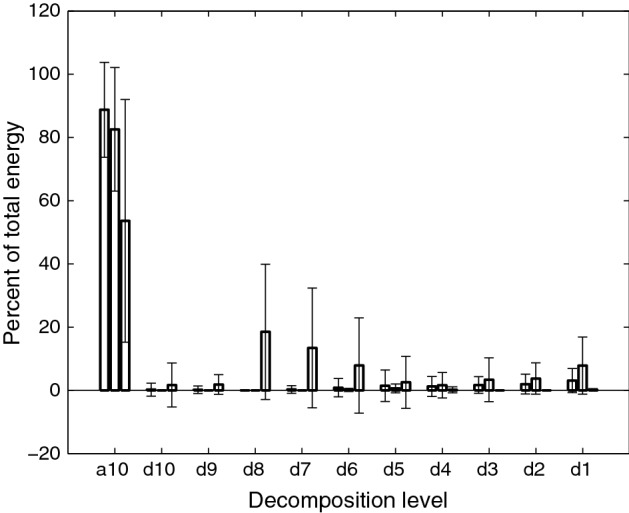
Wavelet energy composition of swallowing vibrations and sounds during viscous swallows. From left to right, the bars for each decomposition level correspond to the signals recorded from the anterior–posterior accelerometer, the superior–inferior accelerometer, and the microphone

The device noise filtering algorithm was recalculated with respect to the microphone system and an FIR filter was applied to the swallowing sound signal to whiten the device noise in that signal. We also applied the same 10 level wavelet denoising process to remove the white noise from our sound data. No splines or other low-frequency removal techniques were applied to the swallowing sounds because we had not investigated if such frequencies contained important sound information.

In a clinical environment, the moments that a swallow begins and ends are determined through visual analysis of a concurrent videofluoroscopy exam. However, our healthy data set did not incorporate this imaging technique as these subjects did not have a medical need for such a procedure. We instead utilized a custom segmentation algorithm that has been shown, in an earlier study, to provide similar results for healthy subjects [27]. Previous research by Wang and Willett demonstrated a useful method for segmenting data sets into two distinct categories based on the local variance of the data [28]. We applied a modified version of their method to this study, which utilizes a two-class fuzzy c-means classification algorithm [27]. This algorithm analyzes only the swallowing vibration data and identifies continuous periods of time where swallows are (periods of high variance in the vibration signal) or are not (periods of low variance in the vibration signal) occurring [27]. The timepoints this algorithm produced allowed us to identify what portions of our data directly corresponded to swallows made by our healthy subjects, thereby segmenting our healthy acoustic and vibratory data.

Unlike in the healthy subject study [15], our fuzzy c-means algorithm has not been fully tested on data from non-healthy subjects and so we could not use it to segment that half of our dataset. We instead utilized a concurrent videofluoroscopic analysis that was carried out as part of the examination of our non-healthy subjects. Two judges, both speech language pathologists with dysphagia research experience and whose inter- and intra-rater reliability in the measures used in this study have been established in prior published research, visually inspected the videofluoroscopic data to measure two parameters: the duration of the swallowing segments and the extent of airway penetration or aspiration during the swallowing segments using the penetration aspiration scale [29]. One of these judges is a co-developer of the penetration aspiration scale who developed decision-making rules for selection of specific frames marking segment duration onset and offset and in rating of the extent of airway protection during the swallow using the eight-point penetration-aspiration scale. They then trained the second judge in methods of selection of these video frames. After training, both judges evaluated a set of twenty-five unfamiliar video recorded swallows, none of which were included in the participant data for the present study. Judgment reliability was evaluated using the intraclass correlation coefficient. The intra-rater and inter-rater intraclass correlation coefficients were both 0.998. Following establishment of acceptable intra- and inter-rater reliability for segment durations and penetration-aspiration scores, the second judge then evaluated the segment onset, segment offset, and penetration-aspiration scale scores for each swallow described in the present study.

Blinded to the recorded signals, these judges segmented and labeled each individual swallow. The beginning (onset) of a swallow segment was defined as the time at which the leading edge of the swallowed bolus intersected with the shadow cast on the x-ray image by the posterior border of the ramus of the mandible while the end (offset) was the time at which the hyoid bone completed motion associated with swallowing-related pharyngeal activity and returned to its resting or pre-swallow position. The time points provided by this procedure were used to segment the vibratory and acoustic signals, thereby obtaining data corresponding to individual non-healthy swallows. Each swallow was also rated on a standard 8-point ordinal clinical penetration-aspiration scale (PA scale) [29] and any swallows with a rating of 3 or lower were included in our analysis as a non-aspirating swallow. Scores of 3 or lower on this scale indicate that either no material entered the upper airway (score of 1), or shallow penetration of the larynx without (score of 2) or with (score of 3) some residue of swallowed material remaining in the larynx after the swallow. Our cut-off point for penetration-aspiration scores was chosen to equalize the severity of airway protection between healthy and disordered participants and minimize the effect of confounding variables, as these scores are common among more elderly patients even without dysphagia [30]. Deeper laryngeal penetration, and especially aspiration into the trachea, represented by scale scores of 4 and higher, have been found to occur with negligible frequency in healthy persons and so would not be a reasonable comparison to our healthy data set [30, 31].

### Feature extraction

Once the signals were filtered and segmented we calculated several different mathematical features in order to characterize each swallow. Our feature selection (the details of which are reproduced below for convenience) once again mirrored the selection used in our previous work that exclusively analyzed healthy subjects [15], so that we could compare our healthy and non-healthy data sets fairly. None of these features are explicitly designed to analyze swallowing signals. Nonetheless, we feel that they are applicable to most signals and should provide a generalized overview of these signals’ traits as they have for similar preliminary studies [20, 32–34].

In the time domain, we investigated the skewness and kurtosis of the signal, which can be calculated with the typical statistical formulas [35]. We also calculated multiple information-theoretic features. The signals were first normalized to zero mean and unit variance, then quantized into ten equally spaced levels, ranging from zero to nine, that contained all recorded signal values. We then calculated the entropy rate feature of the signals. This value is found by subtracting the minimum value of the normalized entropy rate of the signal from 1 to produce a value that ranges from zero, for a completely random signal, to 1, for a completely regular signal [20, 36, 37]. The normalized entropy rate (*NER*) is calculated as

1 $$\begin{aligned} NER(L)=\dfrac{SE(L)-SE(L-1)+SE(1)*perc(L)}{SE(1)} \end{aligned}$$

where *perc* is the percent of unique entries in the given sequence *L* [20]. *SE* is the Shannon entropy of the sequence and is calculated as

2 $$\begin{aligned} SE(L)=-\sum \limits _{j=0}^{10^{L}-1}\rho (j)\ln (\rho (j)) \end{aligned}$$

where $\rho (j)$ is the probability mass function of the given sequence. Quantizing the original signal to 100 discrete levels instead of ten allowed us to calculate the Lempel–Ziv complexity (*LZC*) as

3 $$\begin{aligned} LZC=\dfrac{k\log _{100}n}{n} \end{aligned}$$

where *k* is the number of unique sequences in the decomposed signal and *n* is the pattern length [38].

We also investigated several features in the frequency domain. The center frequency (*C*), sometimes referred to as the spectral centroid, was simply calculated by taking the Fourier transform of the signal and finding the weighted average of all the positive frequency components:

4 $$\begin{aligned} C=\dfrac{\sum \limits _{n=0}^{N-1}f(n)x(n)}{\sum \limits _{n=0}^{N-1}x(n)} \end{aligned}$$

where *x*(*n*) is the magnitude of a frequency component and *f*(*n*) is the frequency of that component. Similarly, the peak frequency was found to be the Fourier frequency component with the greatest spectral energy. We defined the bandwidth of the signal as the standard deviation of its Fourier transform [20].

Lastly, we characterized our signal in the time–frequency domain. Previous contributions found that swallowing signals are to some degree non-stationary [39], to which wavelet decomposition is better suited than a simple Fourier analysis [40]. We chose to decompose our signal using tenth-order Meyer wavelets because they are continuous, have a known scaling function, and more closely resemble swallowing signals in the time domain compared to Gaussian or other common wavelet shapes [26]. The energy in a given decomposition level was defined as

5 $$\begin{aligned} E_{x}=||x||^{2} \end{aligned}$$

where *x* represents a vector of the approximation coefficients or one of the vectors representing the detail coefficients. $||*||$ denotes the Euclidean norm [20]. The total energy of the signal is simply the sum of the energy at each decomposition level. From there, we could calculate the wavelet entropy (*WE*) as:

6 $$\begin{aligned} WE=-\dfrac{Er_{a_{10}}}{100}\log _{2}{\dfrac{Er_{a_{10}}}{100}}-\sum \limits _{k=1}^{10}\dfrac{Er_{d_{k}}}{100}\log _{2}{\dfrac{Er_{d_{k}}}{100}} \end{aligned}$$

where *Er* is the relative contribution of a given decomposition level to the total energy in the signal and is given as [20]

7 $$\begin{aligned} Er_{x}=\dfrac{E_{x}}{E_{total}}*100\% \end{aligned}$$

### Statistical analysis

After calculating the relevant features we performed four sets of statistical comparisons on our data set. Since we only compared identical signals with respect to our chosen variables (i.e. anterior–posterior signals from one group are compared only to the anterior–posterior signals from the other group) and each of our three signals have nine descriptive features, we used a ‘statistical family’ size of 27 for the relevant corrections. First, we used the Wilcoxon rank sum test to non-parametrically test for differences with regards to each feature of all three signals for swallows made by healthy people and swallows made by patients with dysphagia but without stroke. We used the common null hypothesis that the distribution of features in both groups are statistically similar. In this situation, data were separated based on the consistency of the ingested bolus and a p-value of 0.002 was used to determine significance after applying the Bonferroni correction to a standard p-value of 0.05. This process was repeated to test for differences between non-healthy patients with and without stroke. To mirror the results of our previous study we performed a third set of rank sum tests to examine sex-based differences in the data recorded from the non-healthy population. The data were separated based on the presence or absence of stroke and the Holm–Bonferroni correction was applied with a starting p-value of 0.05. The different correction factors were applied due to the expected effect of a given trait on the signal. For example a preliminary analysis showed that the sex of the test subject had a low impact on the recorded signal, and so the more computationally complex but less conservative correction factor was used.

Finally, the effects of bolus viscosity on our data were examined through the use of Wilcoxon signed-rank tests. Again, the data were analyzed separately based on the presence or absence of stroke and the Holm–Bonferroni correction was applied. The null hypothesis remained unchanged. Table 1 summarizes our statistical strategy. The age of the subjects was not utilized as a variable for any of our statistical tests since previous work has shown little significant effect of age on cervical auscultation signals even for large age differences [41].

**Table 1:** Summary of statistical tests

Statistical test	Population	Between-groups variable	Within-groups variable	Correction
Rank-sum	Any non-stroke	Presence of dysphagia	Bolus consistency	Bonferroni ($p=0.002$)
Rank-sum	Any non-healthy	Presence of stroke	Bolus consistency	Bonferroni ($p=0.002$)
Rank-sum	Any non-healthy	Participant’s sex	Presence of stroke	Holm–Bonferroni ($p=0.05$)
Sign-rank	Any non-healthy	Bolus viscosity	Presence of stroke	Holm–Bonferroni ($p=0.05$)

Post hoc estimates of our statistical power were carried out in the GPower software program [42]. We used Lehmann’s method of estimation with a target power of at least 0.80. In mathematical form:

8 $$\begin{aligned} power = 1-\Phi (\frac{c-E(W)}{\sqrt{Var(W)}}) \end{aligned}$$

where *c* is the critical value of the test statistic and is equal to 1.64, *E*() and *Var*() are the expected value and variance operators, respectively, and $\Phi$ is the normal cumulative distribution function. *W* is the Mann–Whitney statistic and is the number of instances where a data point from one group has a lower rank than the data points in the alternate group. We found that our comparisons of normal and non-healthy populations had a sufficient number of swallows to identify a relatively small ($d=0.23$) effect size according to standard conventions [42]. Our remaining tests, due to having fewer samples in each group, only had sufficient power to differentiate moderately larger ($d=0.41$) effects.

## Results

Tables 2, 3, 4, 5 present the mean and standard deviation of each feature of our data set separated by bolus viscosity. Values for these features corresponding to healthy subjects can be found in our previous work [15].

Comparing data from this study collected from patients with dysphagia but without stroke to data collected in our previous study from healthy subjects [15] found many significant differences. For thin swallows, the non-healthy population data demonstrated greater Lempel–Ziv complexity, center frequency, peak frequency, and bandwidth for all three signals ($p<<0.001$ for all) while demonstrating lower kurtosis, entropy rate, and wavelet entropy ($p<<0.001$ for all). The skewness of the data was mixed. It was lower in magnitude for the anterior–posterior accelerometer signal ($p<<0.001$), but higher in magnitude for the superior–inferior signal as well as the microphone signal ($p<<0.001$ for both) in those subjects suspected of having dysphagia. The viscous swallows demonstrated fewer differences between the healthy and non-healthy populations. As with the thin swallows, the viscous non-healthy swallows exhibited greater Lempel–Ziv complexity as well as lower entropy rate and wavelet entropy for all three signals ($p<<0.001$ for all). However, only the anterior–posterior accelerometer signal demonstrated greater skewness, center frequency, and bandwidth as well as lower kurtosis ($p<<0.001$ for all) for viscous swallows from non-healthy subjects. Meanwhile the superior–inferior accelerometer signal demonstrated a lower center frequency and bandwidth while the superior–inferior accelerometer and microphone signals demonstrated increased peak frequencies ($p<<0.001$ for all). These results are also seen in Tables 6 and 7.

While normal and non-healthy swallows showed many differences, comparing non-healthy data with and without the presence of stroke resulted in few statistically significant differences. The data from patients with a history of stroke demonstrated a higher center frequency in the anterior–posterior accelerometer signal ($p=0.006$) along with a greater skewness magnitude in the superior–inferior accelerometer signal ($p=0.01$) and greater entropy rate in the microphone signal ($p=0.03$). These results are also seen in Table 8.

**Table 2:** Time domain features for patients with dysphagia performing thin swallows

	Non-stroke			Stroke		
	A–P	S–I	Sounds	A–P	S–I	Sounds
Skewness	0.307 ± 1.800	− 0.087 ± 2.396	0.331 ± 5.253	0.545 ± 2.710	− 1.038 ± 1.751	2.082 ± 9.061
Kurtosis	21.98 ± 29.58	25.18 ± 67.34	342.1 ± 482.3	49.86 ± 152.3	22.54 ± 44.13	523.7 ± 978.5
Entropy rate	0.986 ± 0.006	0.988 ± 0.004	0.987 ± 0.008	0.985 ± 0.009	0.986 ± 0.008	0.989 ± 0.008
L–Z complexity	0.065 ± 0.024	0.073 ± 0.027	0.034 ± 0.018	0.056 ± 0.020	0.065 ± 0.017	0.028 ± 0.019

**Table 3:** Frequency domain features for patients with dysphagia performing thin swallows

	Non-stroke			Stroke		
	A–P	S–I	Sounds	A–P	S–I	Sounds
Peak frequency (Hz)	11.68 ± 27.98	11.74 ± 14.58	304.0 ± 491.0	34.54 ± 97.57	14.94 ± 54.28	257.0 ± 433.4
Center frequency (Hz)	73.15 ± 113.9	54.60 ± 84.64	801.9 ± 682.6	199.3 ± 291.0	90.75 ± 184.6	895.9 ± 899.3
Bandwidth (Hz)	134.75 ± 211.4	92.45 ± 106.8	552.1 ± 562.0	344.2 ± 498.2	136.4 ± 265.6	697.3 ± 704.4
Wavelet entropy	0.905 ± 0.703	1.063 ± 0.707	1.185 ± 0.725	1.204 ± 0.870	1.171 ± 0.772	1.027 ± 0.776

Our results continued to demonstrate statistical significance when grouped by the sex of the participants. For non-healthy patients without stroke males demonstrated greater skewness magnitude ($p=0.015$) but lower kurtosis ($p=0.020$) for the anterior–posterior accelerometer only. For non-healthy patients with stroke, our data showed significantly greater Lempel–Ziv complexity ($p=0.013$) and bandwidth ($p=0.003$) in the anterior–posterior accelerometer signal for male participants while the center frequency ($p=0.018$) and wavelet entropy ($p=0.005$) for the anterior–posterior accelerometer signal and the entropy rate of the superior–inferior accelerometry signal ($p=0.005$) were lower in the male population. These results are also seen in Tables 9 and 10.

**Table 4:** Time domain features for patients with dysphagia performing viscous swallows

	Non-stroke			Stroke		
	A–P	S–I	Sounds	A–P	S–I	Sounds
Skewness	0.414 ± 1.126	− 0.350 ± 1.684	− 0.454 ± 6.055	− 0.440 ± 3.314	− 0.050 ± 1.078	1.083 ± 2.478
Kurtosis	14.88 ± 28.27	13.80 ± 14.75	426.9 ± 965.2	43.32 ± 147.3	10.42 ± 13.04	281.2 ± 468.1
Entropy rate	0.988 ± 0.005	0.988 ± 0.005	0.990 ± 0.006	0.988 ± 0.006	0.988 ± 0.005	0.991 ± 0.005
L–Z complexity	0.068 ± 0.021	0.073 ± 0.025	0.033 ± 0.018	0.060 ± 0.028	0.072 ± 0.024	0.029 ± 0.018

**Table 5:** Frequency domain features for patients with dysphagia performing viscous swallows

	Non-stroke			Stroke		
	A–P	S–I	Sounds	A–P	S–I	Sounds
Peak frequency (Hz)	10.53 ± 22.95	10.02 ± 12.65	64.03 ± 217.5	21.07 ± 54.81	19.55 ± 48.17	59.36 ± 199.0
Center frequency (Hz)	93.42 ± 301.3	32.27 ± 23.70	850.4 ± 1289	132.5 ± 315.1	34.34 ± 67.53	788.5 ± 1242
Bandwidth (Hz)	202.7 ± 557.1	63.60 ± 68.09	615.3 ± 762.6	283.6 ± 518.1	82.99 ± 116.7	666.4 ± 824.2
Wavelet entropy	0.625 ± 0.637	0.946 ± 0.693	0.908 ± 0.786	0.568 ± 0.610	0.719 ± 0.545	0.801 ± 0.794

**Table 6:** Statistically significant features (healthy vs non-healthy thin swallows)

Feature	A–P	S–I	Sound
Skewness	$p<<0.001$	$p<<0.001$	$p<<0.001$
Kurtosis	$p<<0.001$	$p<<0.001$	$p<<0.001$
Entropy rate	$p<<0.001$	$p<<0.001$	$p<<0.001$
L–Z complexity	$p<<0.001$	$p<<0.001$	$p<<0.001$
Peak frequency	$p<<0.001$	$p<<0.001$	$p<<0.001$
Center frequency	$p<<0.001$	$p<<0.001$	$p<<0.001$
Bandwidth	$p<<0.001$	$p<<0.001$	$p<<0.001$
Wavelet entropy	$p<<0.001$	$p<<0.001$	$p<<0.001$

Lastly, we found a few significant differences between thin and viscous swallows. For non-stroke patients, the higher viscosity bolus produced lower kurtosis ($p=0.005$) and wavelet entropy ($p=0.019$) for the anterior–posterior accelerometer signal along with a lower peak frequency ($p=0.024$) and wavelet entropy ($p=0.032$) for the microphone signal. For stroke patients, we found that increasing the viscosity decreased the anterior–posterior center frequency ($p=0.028$), microphone peak frequency ($p=0.023$), anterior–posterior wavelet entropy ($p=0.011$), and superior–inferior wavelet entropy ($p=0.029$). These results are also seen in Tables 11 and 12.

Figures 2 and 3 show the mean and standard deviation of the energy distribution of the wavelet decomposition of all three signals for thin and viscous swallows, respectively, from non-healthy subjects. The x-axis of these figures represents the time–frequency bands of the decomposition, with d1 being the highest (5–10 kHz) and a10 being the lowest (0–10 Hz), while the y-axis indicates the percent of the signal’s energy that is contained within that frequency band. The vibrations demonstrate similar behavior to those observed in our previous studies, with the majority of energy being present in the lowest frequency level [15, 41]. The swallowing sounds, however, demonstrate a large increase in energy in the d8 through d6 bands (corresponding to approximately 40–300 Hz in this study) that was not present in our earlier findings [15, 41]. No statistical tests were used to analyze these decompositions because the wavelet entropy feature already provides a holistic summary of this information.

**Table 7:** Statistically significant features (healthy vs non-healthy viscous swallows)

Feature	A–P	S–I	Sound
Skewness	$p<<0.001$	–	–
Kurtosis	$p<<0.001$	–	–
Entropy rate	$p<<0.001$	$p<<0.001$	$p<<0.001$
L–Z complexity	$p<<0.001$	$p<<0.001$	$p<<0.001$
Peak frequency	–	$p<<0.001$	$p<<0.001$
Center frequency	$p<<0.001$	$p<<0.001$	–
Bandwidth	$p<<0.001$	$p<<0.001$	–
Wavelet entropy	$p<<0.001$	$p<<0.001$	$p<<0.001$

**Table 8:** Statistically significant features (stroke vs non-stroke)

Feature	A–P	S–I	Sound
Skewness	–	$p=0.01$	–
Entropy rate	–	–	$p=0.03$
Center frequency	$p=0.006$	–	–

**Table 9:** Statistically significant features (male vs female non-stroke)

Feature	A–P	S–I	Sound
Skewness	$p=0.015$	–	–
Kurtosis	$p=0.020$	–	–

## Discussion

Our study found many differences between acoustic and vibratory signals recorded during swallows produced by healthy and non-healthy patients. When performing thin swallows, subjects with dysphagia demonstrated higher frequency sounds and vibrations with greater Lempel–Ziv complexity, but lower kurtosis, entropy rate, and wavelet entropy. Similar results were found when comparing swallows made with viscous liquid, but in this case the statistical significance of the swallowing sounds and superior–inferior vibrations were lost with respect to skewness, kurtosis, and center frequency. Together, these factors all indicate a signal that contains more, sudden changes in intensity with less predictability. We chose to rule out the possibility that administering different brands of test liquids caused these results due to the viscosity information gathered (via repeated measures) in both this and our previous study [15] indicating that the two brands provide similarly viscous products. Additionally, if the different brands were to blame, then we would expect the data from non-healthy patients to give results opposite to those shown as the Varibar Thin Liquid is known to be slightly more viscous than ordinary water. However, we are still unsure as to what underlying mechanics did result in this reported variation between healthy and non-healthy swallows. Since we did not control for the original cause of dysphagia, with the exception of stroke patients, the signal variations we observed could represent different pathophysiological effects caused by the various disease-related causes of dysphagia. We suspect that our data could be indicative of pathology-specific impairments in hyolaryngeal movement during a swallow, as this motion contributes significantly to swallowing vibrations [32], but we have no further evidence to support this point at this time.

In contrast to our previous work on this subject with healthy patients [15, 41], the data gathered from non-healthy patients showed few significantly different features with respect to the subject’s sex. Also, some of the differences that are present appear to be counter-intuitive, such as how males with stroke showed decreased anterior–posterior center frequency but greater bandwidth than women. This implies that, rather than having similar frequency distributions that are shifted in one direction or the other, swallows made by non-healthy males and females have completely different frequency distributions. However, we did note that the distribution of feature values was notably wider for non-healthy subjects than those without dysphagia [15]. We feel that this is indicative of the increased perturbation of swallowing function in patients with dysphagia as well as etiological differences in the pathophysiology of each patient’s swallowing disorder. Even if two patients receive the same diagnosis they may express different symptoms or severity of those symptoms due to a variety of neurological and other disease-specific factors. For example, two patients may experience a stroke and have difficulties swallowing as a result, but the location and size of the lesion will affect their overall sensori-motor functions and can result in a ‘personalized’ form of dysphagia [2]. As a result, we conclude that the increased feature variability is greater than and may mask any effect that the patient’s sex has on the data recorded from non-healthy subjects.

**Table 10:** Statistically significant features (male vs female stroke)

Feature	A–P	S–I	Sound
Entropy rate	–	$p=0.005$	–
L–Z complexity	$p=0.013$	–	–
Center frequency	$p=0.018$	–	–
Bandwidth	$p=0.003$	–	–
Wavelet entropy	$p=0.005$	–	–

**Table 11:** Statistically significant features (thin vs viscous non-stroke)

Feature	A–P	S–I	Sound
Kurtosis	$p=0.005$	–	–
Peak frequency	–	–	$p=0.024$
Wavelet entropy	$p=0.019$	–	$p=0.032$

**Table 12:** Statistically significant features (thin vs viscous stroke)

Feature	A–P	S–I	Sound
Peak frequency	–	–	$p=0.023$
Center frequency	$p=0.028$	–	–
Wavelet entropy	$p=0.011$	$p=0.029$	–

Though we did not find as many statistical differences for non-healthy patients as we did for the healthy cohort [15], our examination of the effects of fluid viscosity are similar to what was expected. For non-healthy patients both with and without stroke we see that swallowing higher viscosity fluid produced sounds and vibrations with lower frequency, kurtosis, and entropy. Again, for much the same logic as to why we observed fewer effects of the patient’s sex, we feel that the lower number of features demonstrating statistical significance is a result of the highly variable nature of dysphagia.

The increase in wavelet energy for swallowing sounds in the 40–300 Hz range may or may not be a clinically significant finding. The initial, and perhaps most likely, consideration is that this range corresponds to the frequency of electrical power transmission along with several higher harmonics. Since any x-ray camera will have a significant power draw when it is under operation, it is possible that the wiring for our microphone picked up this radiation during the experiment and corrupted our signal to some degree. However we did not see a similar spike in energy for the signals recorded by the accelerometer, which is not as well shielded from such interference. One also cannot help but notice that this range also corresponds to the reported range of the fundamental frequency of human vocal folds during speech related phonation [43, 44]. Approximately half of the swallows made by non-healthy subjects and included in this study rated a level of 2 or 3 on the penetration-aspiration scale, which indicated that the laryngeal vestibule was either not completely closed at the time the swallowed material was present in the lower pharynx or did not close in a timely manner. Such behavior is commonly seen as a result of the many functional or structural forms of dysphagia and enables shallow penetration of the bolus into the airway [30]. However, shallow laryngeal penetration is not uncommon across the age spectrum [30, 45]. It is at least theoretically possible that the pressure changes during a swallow, combined with an incomplete sealing of the laryngeal vestibule, forces air across the vocal folds and produces sounds which would never occur in a healthy subject whose larynx is completely closed during the swallow. Another explanation might be that in some pathological states the coordination of swallowing and breathing is affected with reversal of the typical exhale-swallow-exhale pattern predominating [46–48]. Again, the lack of similar results for swallowing vibrations is perplexing, but we believe that this issue could merit further investigation in the future.

Our study found only 3 out of 27 statistically different features with respect to the sounds and vibrations produced by patients with stroke and those produced by patients with other causes of dysphagia. This implies one of three possibilities. The first is that, despite other differences, dysphagia as a symptom of a stroke is functionally equivalent to dysphagia as a symptom of another condition and produces the same sound and vibration pattern. The second, and seemingly much more likely option given the voluminous studies demonstrating distinctly different disease-specific patterns of swallow kinematics, is that dysphagia as a symptom of stroke does not result in any reliable and consistent alterations to our chosen signals. Instead, the feature values of our signals may vary a great deal but not in such a way to make the population distribution significantly higher or lower than the non-stroke population. Lastly, these results may simply be due to a small sample size of patients with stroke and that we were unable to properly estimate the true distribution. However, as each patient made multiple swallows over the course of their examination, we believe that the effect of our population size is minimized. Judging by the high standard deviation of all of our chosen features and previously described variable nature of dysphagia, we believe that the second option is the most likely cause of our reported results.

## Implications and Limitations

It is important to note that the conclusions drawn from this study are based exclusively on our selection of features. These mathematical features were chosen, not necessarily because they are known to characterize swallowing sounds and vibrations well, but because they are rather generalized and can be applied reasonably effectively to most signals. Though our results can be easily compared to other studies or assessed intuitively, we lose a certain amount of precision and statistical power to do so. It should be noted, however, that this is not entirely a design decision. At the time of this writing, no consensus has been reached on what the key features of a cervical auscultation signal are. Past research has shown the usefulness of these features with regards to characterization and classification [20, 32–34], but they have not been widely adopted in the field. We chose to use a broad selection of generalized, multi-domain features in order to take advantage of this past work and minimize the risk of producing overly-specific results. From this foundation, we can provide insights as to what more complex, and potentially better suited, features would or would not be beneficial to investigate in future studies.

We also make note of the narrow selection of swallows we utilized for this study, specifically thin or somewhat viscous liquid swallows made in a neutral head position. In order to minimize the number of variables involved in a single study we did not analyze different head positions or swallowing strategies, more varied liquid or solid boluses, or boluses of greater or smaller volumes. All of these variables are known to have an effect on swallowing performance, but their relation to cervical auscultation signals is unclear and was not investigated in this study.

We believe that our results help to further reveal the nature of cervical auscultation signals. By providing a simple baseline that can be used to generalize the differences between signals recorded from healthy and non-healthy subjects, it should be easier to develop more specialized analysis methods. For example, many of our simple frequency domain features demonstrated statistically significant differences between healthy and non-healthy subjects, which indicates that an analysis method which examines the relative strength of several frequency components in greater detail could be of benefit. Likewise, our results provide a general guide as to what frequency components would be of greatest interest and help to simplify a high-dimensional problem.

It is also possible that the results of this study could be utilized in a more direct manner for differentiating normal and abnormal swallows. This study has demonstrated that the value of many cervical auscultation features change based on the condition of the subject. If the differences are of a sufficient magnitude, our findings could be used to develop a novel classification technique to differentiate healthy and non-healthy patients in a clinical setting. Such a technique would add a degree of objectivity and automation to otherwise subjective clinical swallowing screenings.

## Conclusion

In this study, we sought to characterize how swallowing sounds and vibrations differ between healthy subjects and subjects with dysphagia using a broad selection of generalized statistical features. We found that, for non-aspirating swallows, the majority of our chosen features did show significant differences between these two groups for both sounds and vibrations. We were also able to confirm our previous findings on the effects of fluid viscosity and the subject’s sex on our chosen features. Finally, we found extremely few differences in our chosen signal features between patients with dysphagia as a symptom of stroke and patients with other causes of dysphagia, indicating that dysphagia due to stroke does not result in a single, well-defined functional change. These findings should greatly help the development of the cervical auscultation field and serve as a reference for future investigations into more specialized characterization methods.
